# Preoperative SGLT2 Inhibitor Use and Outcomes After Major Cardiac Procedures: A Propensity Score-Matched Analysis of the TriNetX Network

**DOI:** 10.3390/jcm15176808

**Published:** 2026-09-02

**Authors:** Mirza Muhammad Hadeed Khawar, Faizan Ahmed, Mohab Elnashar, Cheryl Vanessa Lewis, Minahil Shahid, Ali Asgher Shuja, Fatima Afzal, Naheed Sajjad, Muhammad Amir Khan, Meeran Atta, Umair Arshad, Muneeb Khawar, Nazila Dalir, Haris Bin Tahir

**Affiliations:** 1 Services Institute of Medical Sciences, Lahore 54000, Pakistan; mirzahadeed64@gmail.com (M.M.H.K.); meeranatta3@gmail.com (M.A.); umairarshadgil@gmail.com (U.A.); 2 Hackensack Meridian Health, Jersey Shore University Medical Center, Neptune, NJ 07753, USA; faizan.ahmed1@hmhn.org; 3 Department of Medicine, University of Arkansas for Medical Sciences, Little Rock, AR 72205, USA; mohabelnashar1998@gmail.com; 4 Department of Medicine, Tbilisi State Medical University, Tbilisi 0186, Georgia; vanessacheryl19@gmail.com; 5 Department of Medicine, Shalamar Medical and Dental College, Lahore 54840, Pakistan; minahilshahid0@gmail.com; 6 Department of Basic Medical Sciences, Faculty of Pharmacy, Salim Habib University, Karachi 74900, Pakistan; ali.asgher@shu.edu.pk; 7 Pharmacy Department, Khalida Memorial Institute of Allied & Medical Sciences, Sialkot 51310, Pakistan; fatima.afzal47@yahoo.com; 8 Department of Medicine, Sardar Bahadur Khan Women University, Quetta 87300, Pakistan; naheedsaj@gmail.com; 9 Department of Medicine, Fergana Medical Institute of Public Health, Fergana 150100, Uzbekistan; amirwazir568@gmail.com; 10 Department of Medicine, King Edward Medical University, Lahore 54000, Pakistan; muneebb.khawar@gmail.com; 11 School of Medicine, St. George’s University, St. George’s 00000, Grenada; 12 Lahore General Hospital, Lahore 54000, Pakistan

**Keywords:** SGLT2 inhibitors, cardiac surgery, transcatheter aortic valve replacement (TAVR), postoperative mortality, acute kidney injury, propensity score matching, TriNetX, real-world evidence

## Abstract

**Background/Objectives:** Sodium–glucose cotransporter-2 (SGLT2) inhibitors improve cardiorenal outcomes in patients with diabetes, heart failure, and chronic kidney disease. Their association with perioperative outcomes after major cardiac procedures remains uncertain. **Methods:** In this retrospective cohort study using the TriNetX U.S. Collaborative Network, adults (≥18 years) undergoing cardiac procedures were identified. Patients with documented SGLT2 inhibitor use within 1 year before the index procedure were compared with non-users. Risk ratios (RR), risk differences, Kaplan–Meier survival curves, and Cox models were used for analysis. **Results:** Of 150,724 patients identified, 7526 propensity score-matched pairs were analyzed. The matched cohorts had a mean age of 65.8 ± 10.6 years and were predominantly male (70.5% male, 29.5% female). Preoperative SGLT2 inhibitor use was associated with significantly lower all-cause mortality at 30 days (1.3% vs. 2.3%; RR 0.566 [95% CI 0.443–0.722], *p* < 0.001), 90 days (2.2% vs. 3.4%; RR 0.643, *p* < 0.001), and 1 year (3.8% vs. 5.8%; RR 0.657, *p* < 0.001). MACE rates were similar between groups at all time points. Acute kidney injury at 30 days was lower with SGLT2 inhibitors (15.4% vs. 17.7%; RR 0.870, *p* < 0.001), whereas hospital admission rates were higher (76.4% vs. 68.7%; RR 1.111 [95% CI 1.089–1.133], *p* < 0.001). These associations were consistent in diabetes-only and obesity-only subgroups and in a 30-day landmark sensitivity analysis. **Conclusions:** In this large, contemporary, multi-institutional propensity score-matched cohort, preoperative SGLT2 inhibitor use was associated with reduced all-cause mortality and acute kidney injury after major cardiac procedures. These real-world findings support the conduct of dedicated randomized trials evaluating perioperative SGLT2 inhibitor therapy.

## 1. Introduction

Major cardiac procedures, including coronary artery bypass grafting (CABG), surgical aortic or mitral valve replacement or repair, transcatheter aortic valve replacement (TAVR), and interventions on the thoracic aorta, continue to carry substantial perioperative and intermediate-term risk despite progressive improvements in surgical technique, anesthesia, and postoperative care [1,2]. Contemporary series still report non-negligible rates of all-cause mortality, major adverse cardiovascular events (MACE), acute kidney injury (AKI), and unplanned hospital admission, each of which contributes meaningfully to patient morbidity, prolonged length of stay, and healthcare resource utilization [3,4]. AKI alone complicates 15–30% of cardiac surgical procedures and remains one of the strongest independent predictors of both short-term and long-term mortality [3,5].

The typical patient undergoing these operations is characterized by a high burden of comorbidities, type 2 diabetes mellitus, heart failure across the ejection-fraction spectrum, chronic kidney disease, hypertension, and obesity, conditions that themselves amplify perioperative risk. Sodium–glucose cotransporter-2 (SGLT2) inhibitors have transformed the management of these comorbidities. Large randomized trials have established that dapagliflozin and empagliflozin reduce the composite of cardiovascular death or heart-failure hospitalization in patients with reduced or preserved ejection fraction and slow the progression of chronic kidney disease, irrespective of diabetes status [6,7,8]. These cardiorenal benefits are mediated by a combination of hemodynamic, metabolic, anti-inflammatory, and anti-fibrotic effects that are particularly relevant in the perioperative setting [9,10].

Recent observational studies and meta-analyses have begun to explore whether these advantages extend to patients undergoing TAVR, with several reports suggesting lower rates of mortality and heart-failure events among SGLT2-inhibitor users [11,12,13,14]. However, data examining preoperative SGLT2-inhibitor exposure across the wider spectrum of major cardiac procedures, CABG, surgical valve interventions, TAVR, and thoracic aortic repair remain limited. No large-scale, multi-institutional analysis has yet evaluated the association of preoperative SGLT2-inhibitor use with hard clinical endpoints in this heterogeneous population.

Leveraging the TriNetX U.S. Collaborative Network, we therefore conducted a propensity score-matched cohort study to determine whether documented SGLT2-inhibitor use within the year preceding major cardiac procedures is associated with reduced all-cause mortality (primary outcome), MACE, AKI, hospital admission, and other clinically relevant perioperative events. The findings are intended to generate hypotheses for future randomized trials evaluating the optimal perioperative use of this drug class.

## 2. Materials and Methods

### 2.1. Study Oversight

All authors participated in the manuscript review and confirmed the data’s accuracy and completeness. Since the study used aggregated, de-identified data from a research network database, it was exempt from institutional review board approval. The study was conducted and reported in accordance with the Strengthening the Reporting of Observational Studies in Epidemiology (STROBE) guidelines for cohort studies [15].

### 2.2. Data Source

We utilized the TriNetX Analytics Network, a global federated research platform that compiles anonymized electronic health record data from more than 120 healthcare organizations across 19 countries [16]. This analysis was performed on the U.S. Collaborative Network, which included 66 healthcare organizations at the time of the study.

### 2.3. Study Population and Design

We performed a retrospective cohort study of adults (≥18 years) who underwent major cardiac procedures. Patients were included if they were aged 18 years or older at the time of the index procedure and underwent one of the following: coronary artery bypass grafting using arterial or venous grafts, open aortic or mitral valve replacement or repair, transcatheter aortic valve replacement (TAVR), or thoracic aorta replacement or repair. Patients were excluded if they had a diagnosis of atrial fibrillation or flutter, heart-transplant status, end-stage renal disease, an implantable heart-assist device, or congenital malformations of the circulatory system. Patients with pre-existing atrial fibrillation or flutter were excluded to create a more homogeneous cohort free of baseline arrhythmia that could independently influence postoperative rhythm-related events, hospital resource use, and mortality, even though postoperative atrial fibrillation itself was not a study endpoint.

Patients were categorized into two groups based on SGLT2i use: those with any documented SGLT2 inhibitor medication record (order or dispensing) within 1 year before the index procedure (SGLT2i group) and those without any such record (control group). A one-year preoperative exposure window was selected to capture patients with recent therapeutic exposure while retaining sufficient sample size in a real-world electronic-health-record network; shorter windows would have substantially reduced the exposed cohort. Because TriNetX captures medication orders or dispensations rather than continuous adherence or exact stop dates, it was not possible to determine whether therapy was ongoing up to the day of surgery or had been interrupted according to perioperative guidelines. The term “preoperative use” therefore refers only to documented exposure in the preceding year and does not imply continuation through the day of the procedure.

The index date was defined as the date of the qualifying cardiac procedure. The eligible index procedures occurred between 1 January 2018 and 31 December 2023. Follow-up windows were 30 days, 90 days, and 1 year after the index event. Further details of the query logic are provided in Supplemental Tables S1 and S2.

The cohort combined several distinct cardiac procedures (CABG, surgical valve replacement or repair, TAVR, and thoracic aortic interventions). These procedures differ in baseline risk, perioperative management, and expected outcomes. Procedure type was included among the covariates used to generate the propensity score. However, the distribution of each individual procedure before and after matching was not extracted from the platform and therefore cannot be reported. Calendar year was also not included as a matching variable, so residual temporal confounding related to the more recent adoption of SGLT2 inhibitors cannot be excluded.

### 2.4. Study Outcomes

The primary outcome was all-cause mortality. Secondary outcomes included major adverse cardiovascular events (MACE; composite of all-cause mortality, acute myocardial infarction, and cerebral infarction), hospital admissions (inpatient encounters), stroke, acute kidney injury, hypotension, cardiac arrest, ventricular arrhythmia, and cardioversion. Detailed outcome definitions are provided in Supplemental Table S3. All outcomes were ascertained only after the index procedure date; diagnoses recorded on the index date itself were not counted as postoperative events.

### 2.5. Statistical Analysis

Continuous variables are expressed as mean ± standard deviation (SD) and were compared using independent-sample t-tests. Categorical variables are presented as counts and percentages and were compared using the chi-square test. Covariates included in the propensity score model comprised age, sex, race, diabetes mellitus, hypertension, chronic kidney disease, heart failure, prior myocardial infarction, body-mass index, estimated glomerular filtration rate, hemoglobin A1c, and use of beta-blockers, statins, and angiotensin-converting-enzyme inhibitors, together with procedure type. The full list of matching variables is also provided in Supplemental Table S4.

To adjust for baseline differences between groups, 1:1 propensity score matching (PSM) was performed using a greedy nearest-neighbor algorithm with a caliper of 0.1 pooled standard deviations [17]. A standardized mean difference (SMD) < 0.10 was considered indicative of adequate balance. Risk differences (RD), risk ratios (RR), and 95% confidence intervals were calculated for all outcomes using the Measure of Association analysis. Kaplan–Meier survival curves and Cox proportional hazards models were generated to estimate hazard ratios. The log-rank test was used to compare survival distributions. Statistical significance was defined as a two-sided *p* < 0.05. All analyses were performed on the TriNetX platform.

### 2.6. Subgroup and Sensitivity Analyses

Prespecified subgroup analyses were performed in diabetes-only and obesity-only patients using the same exposure definition, follow-up duration, outcome definitions, and analytic approach, with separate 1:1 PSM conducted within each subgroup. Sensitivity analyses included a 30-day landmark analysis (outcomes assessed from day 31 onward) and evaluation of falsification outcomes (low back pain and acute upper respiratory tract infection). Details of the propensity score matching procedure are provided in Supplemental Table S4. E-values were calculated to quantify the robustness of findings to unmeasured confounding [16] and are reported in Supplemental Table S5. Because multiple secondary endpoints were evaluated, the possibility of type I error inflation should be considered when interpreting individual secondary outcomes; the primary outcome of all-cause mortality was prespecified and is therefore less susceptible to this concern.

## 3. Results

**Figure 1:**
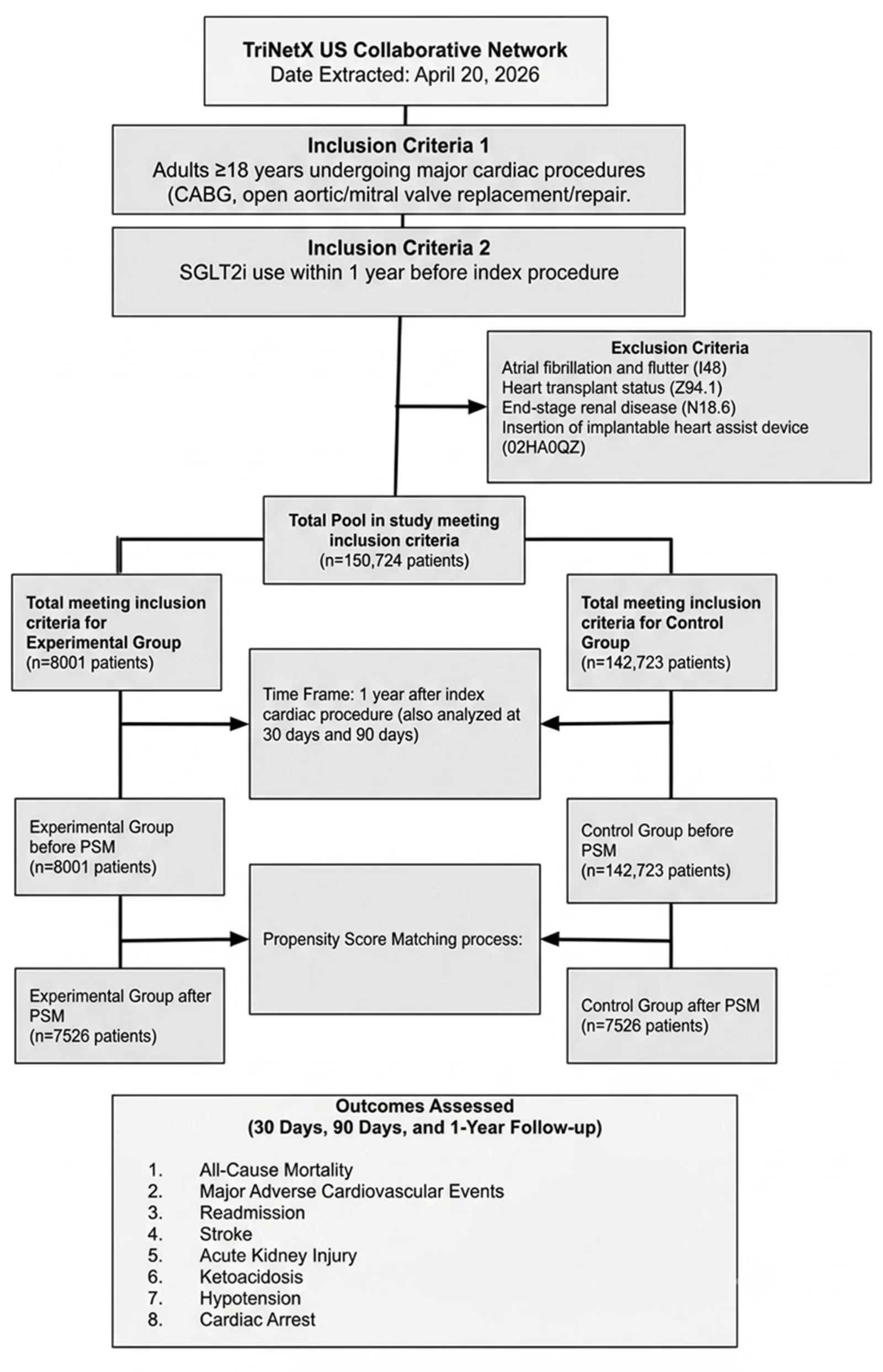
Flow diagram of the study population showing patient identification, exclusion criteria, and 1:1 propensity score matching in the TriNetX U.S. Collaborative Network.

A total of 150,724 patients who underwent qualifying cardiac procedures were identified (Figure 1). After application of the exclusion criteria (atrial fibrillation or flutter, heart-transplant status, end-stage renal disease, implantable heart-assist device, or congenital circulatory malformations) and 1:1 propensity score matching, 7526 patients remained in each cohort.

### 3.1. Patient Characteristics

Baseline characteristics before and after propensity score matching are summarized in Table 1. Prior to matching, SGLT2i users were younger (65.7 ± 10.7 vs. 67.7 ± 12.2 years; SMD 0.169), had a substantially higher prevalence of diabetes mellitus (5858 [76.8%] vs. 38,065 [29.3%]; SMD 1.083), heart failure (4121 [54.0%] vs. 36,035 [27.7%]; SMD 0.555), hypertension (6115 [80.2%] vs. 86,822 [66.9%]; SMD 0.306), and chronic kidney disease (2072 [27.2%] vs. 18,072 [13.9%]; SMD 0.333), and greater use of cardiovascular medications, including beta-blockers (6282 [82.4%] vs. 72,541 [55.9%]; SMD 0.599), statins (6748 [88.5%] vs 78,333 [60.3%]; SMD 0.682), and ACE inhibitors (3162 [41.5%] vs. 34,353 [26.5%]; SMD 0.321). Laboratory values also differed, with higher HbA1c (7.4 ± 1.7% vs. 6.2 ± 1.4%; SMD 0.769) in the SGLT2i group. After propensity score matching, the cohorts were well balanced across all measured demographics, comorbidities, medications, and laboratory values, with standardized mean differences < 0.10 for nearly all covariates.

### 3.2. Study Outcomes

**Figure 2:**
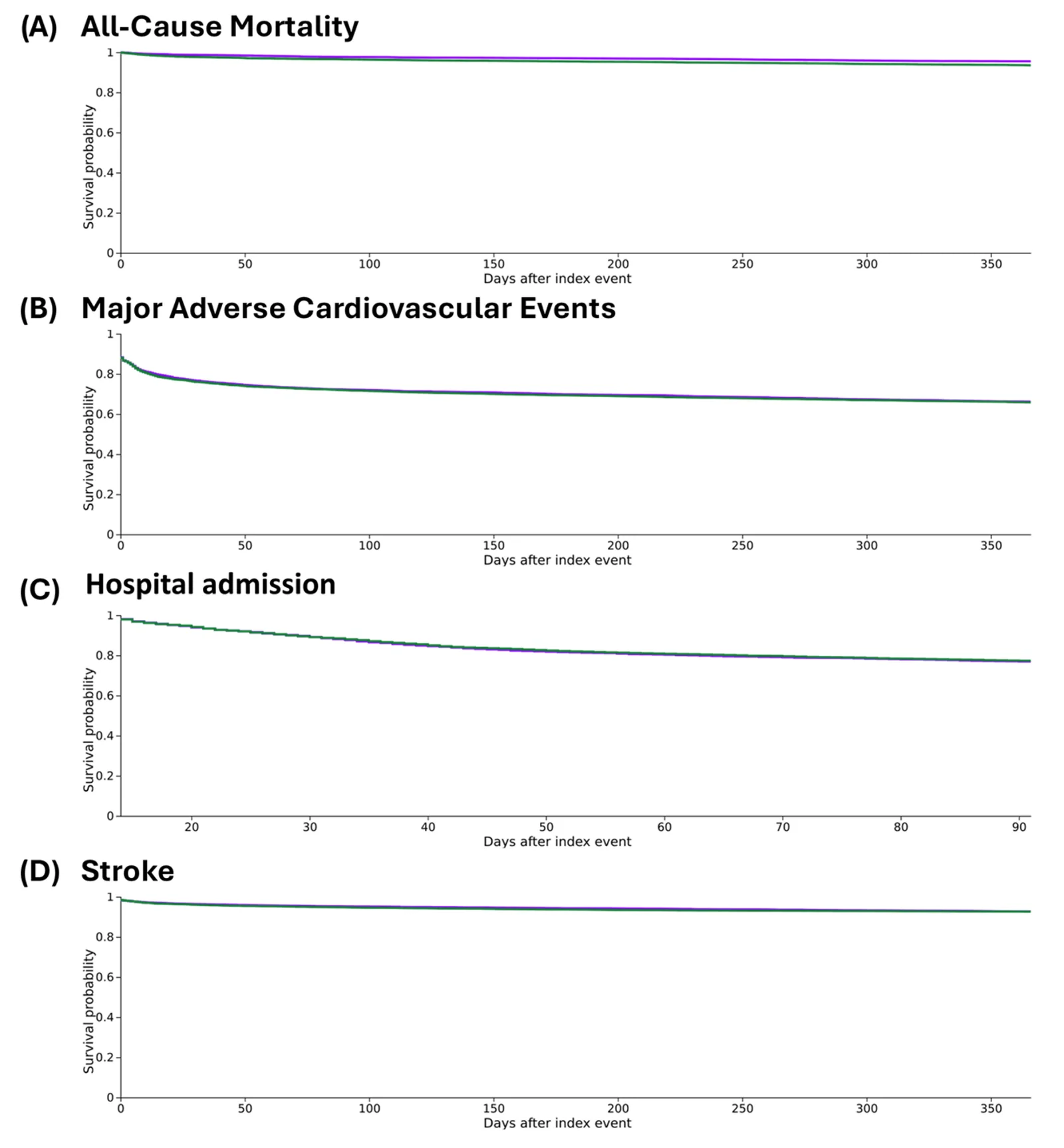
Kaplan–Meier survival curves for (**A**) all-cause mortality, (**B**) major adverse cardiovascular events, (**C**) hospital admission, and (**D**) stroke at 1-year follow-up in propensity score-matched patients with and without preoperative SGLT2 inhibitor use.

**Figure 3:**
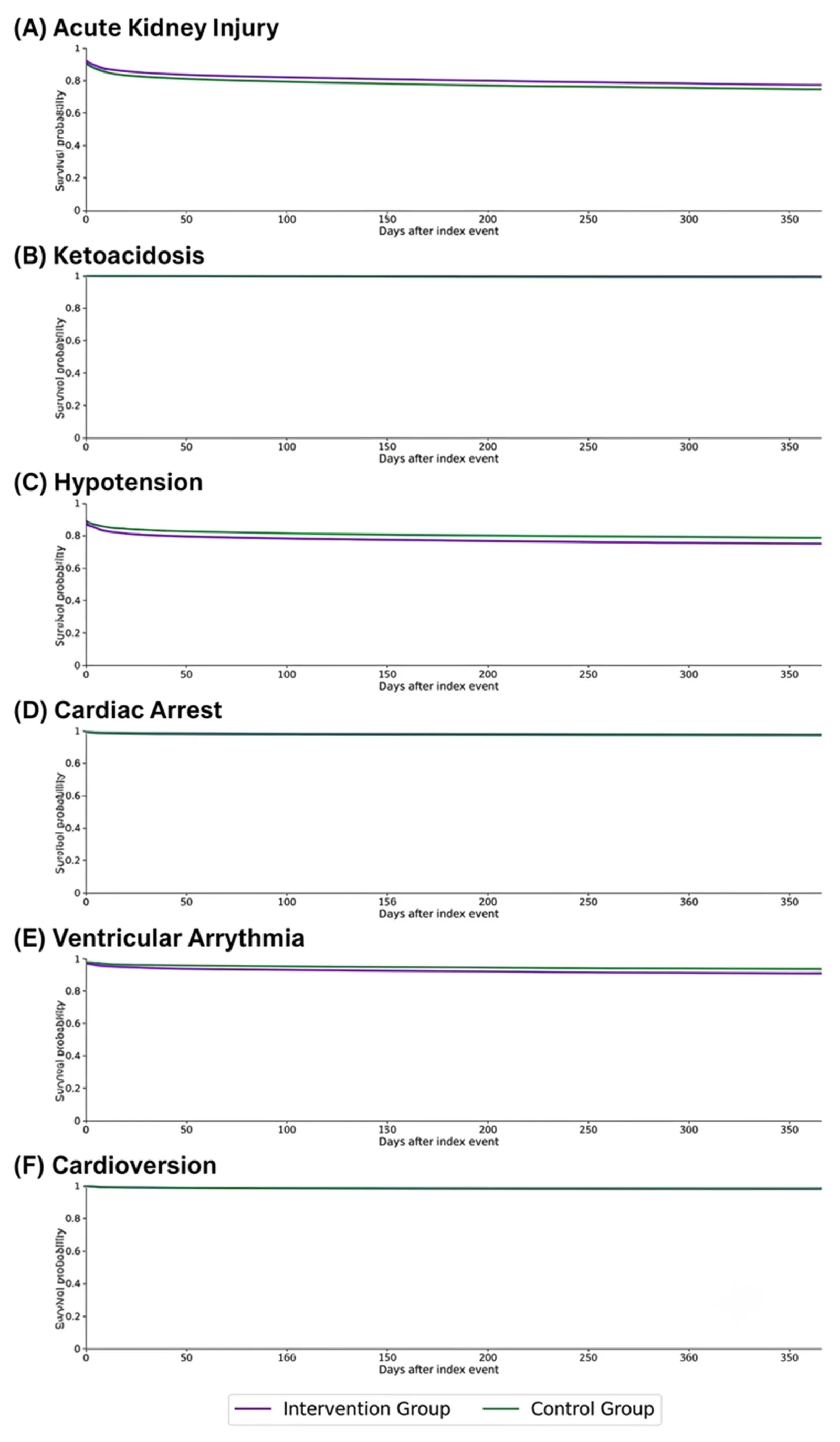
Kaplan–Meier survival curves for (**A**) acute kidney injury, (**B**) ketoacidosis, (**C**) hypotension, (**D**) cardiac arrest, (**E**) ventricular arrhythmia, and (**F**) cardioversion at 1-year follow-up in propensity score-matched patients with and without preoperative SGLT2 inhibitor use.

All outcomes are reported after propensity score matching and are summarized in Table 2 and Figure 2 and Figure 3. SGLT2i use was associated with significantly lower risk of all-cause mortality at 30 days (99 [1.3%] vs. 175 [2.3%]; RD −0.010 [95% CI −0.014 to −0.006]; RR 0.566 [95% CI 0.443–0.722]; *p* < 0.001), 90 days (164 [2.2%] vs. 255 [3.4%]; RD −0.012 [95% CI −0.017 to −0.007]; RR 0.643; *p* < 0.001), and 1 year (285 [3.8%] vs. 434 [5.8%]; RD −0.020 [95% CI −0.027 to −0.013]; RR 0.657; *p* < 0.001). Corresponding hazard ratios from Cox models for all-cause mortality were consistent with the risk-ratio estimates (30-day HR 0.57, 90-day HR 0.64, 1-year HR 0.66; all *p* < 0.001). MACE rates were similar between groups at 30 days (1745 [23.2%] vs. 1795 [23.9%]; RD −0.007 [95% CI −0.020 to 0.007]; RR 0.972; *p* = 0.337), 90 days (2042 [27.1%] vs. 2078 [27.6%]; RD −0.005; RR 0.983; *p* = 0.510), and 1 year (2379 [31.6%] vs. 2449 [32.5%]; RD −0.009; RR 0.971; *p* = 0.222). Because mortality was substantially lower among SGLT2-inhibitor users while the composite MACE rate remained similar, the individual components of MACE (all-cause mortality, acute myocardial infarction, and cerebral infarction) are of particular interest. 

Acute kidney injury was significantly lower with SGLT2i use at 30 days (1157 [15.4%] vs. 1330 [17.7%]; RD −0.023; RR 0.870; *p* < 0.001). Hospital admission rates were higher in the SGLT2i group at 30 days (5748 [76.4%] vs. 5173 [68.7%]; RD 0.076 [95% CI 0.062–0.091]; RR 1.111 [95% CI 1.089–1.133]; *p* < 0.001). Additional 30-day secondary outcomes included lower risk of cardiac arrest (116 [1.5%] vs. 145 [1.9%]; RR 0.800; *p* = 0.070) and higher risk of ventricular arrhythmia (423 [5.6%] vs. 300 [4.0%]; RR 1.410; *p* < 0.001) and hypotension (1463 [19.4%] vs. 1243 [16.5%]; RR 1.177; *p* < 0.001).

### 3.3. Subgroup Analysis

The association of SGLT2i use with lower all-cause mortality was consistent in prespecified diabetes-only and obesity-only subgroups (Table 3). In the diabetes-only subgroup, SGLT2i use was associated with a 34% lower risk of 1-year mortality (RR 0.66 [95% CI 0.55–0.79]; *p* < 0.001). In the obesity-only subgroup, SGLT2i use was associated with a 38% lower risk of 1-year mortality (RR 0.62 [95% CI 0.46–0.84]; *p* = 0.002). Results for other outcomes were directionally consistent with the main analysis.

### 3.4. Sensitivity Analysis

Landmark analysis excluding events within the first 30 days after the index procedure demonstrated consistent protective associations for all-cause mortality and other key outcomes (Table 4). SGLT2i use remained associated with lower risk of mortality from day 31 onward (HR 0.64 [95% CI 0.54–0.75]; *p* < 0.001). Falsification outcomes (low back pain and acute upper respiratory tract infection) showed no significant association with SGLT2i use (detailed risk ratios and confidence intervals are provided in Supplemental Table S5 together with the E-values and their confidence limits).

## 4. Discussion

In this large propensity score-matched analysis of patients undergoing major cardiac procedures in the TriNetX U.S. Collaborative Network, SGLT2 inhibitor use was associated with a significantly lower risk of all-cause mortality at 30 days, 90 days, and 1 year compared with non-users. The association remained consistent in prespecified diabetes-only and obesity-only subgroups and in a 30-day landmark sensitivity analysis. Acute kidney injury was also significantly reduced, whereas MACE rates were comparable between groups and hospital admission rates were modestly higher in the SGLT2i cohort. These findings provide hypothesis-generating evidence that SGLT2 inhibitors may confer cardiovascular and renal protection in a broad real-world population undergoing CABG, surgical valve replacement/repair, TAVR, and thoracic aorta interventions.

The higher 30-day hospital-admission rate observed among SGLT2-inhibitor users (76.4% vs. 68.7%) is unexpected in light of the concurrent reductions in mortality and AKI and warrants careful interpretation. Several non-mutually exclusive explanations are plausible. First, residual confounding by indication or by unmeasured severity of illness may persist; patients prescribed SGLT2 inhibitors often have more advanced cardiorenal disease and may therefore be monitored more intensively or readmitted at a lower threshold. Second, the definition of “hospital admission” in TriNetX encompasses any inpatient encounter, including planned postoperative stays and transfers, which may differ systematically between centers that more frequently prescribe SGLT2 inhibitors. Third, SGLT2-inhibitor-related hemodynamic or volume shifts (or clinician awareness of these effects) could prompt earlier evaluation and admission even when the ultimate clinical course is favorable. Importantly, the excess admissions did not translate into higher mortality or MACE, suggesting that any increase in resource utilization was not accompanied by worse hard outcomes. Dedicated prospective studies with granular data on admission indication and length of stay will be required to clarify this observation.

In addition to the higher hospital-admission rate, SGLT2-inhibitor users also experienced higher rates of ventricular arrhythmia (5.6% vs. 4.0%) and hypotension (19.4% vs. 16.5%) at 30 days. These observations may reflect the known hemodynamic and volume effects of SGLT2 inhibitors, including natriuresis, mild volume depletion, and blood-pressure lowering, which can become clinically apparent in the early postoperative period when fluid shifts and vasoactive medications are common. Alternatively, residual confounding by indication cannot be excluded, as patients prescribed SGLT2 inhibitors may have had more advanced heart failure or higher arrhythmic risk at baseline. Importantly, the excess of these intermediate events did not translate into higher rates of cardiac arrest or mortality, suggesting that they were either transient or successfully managed. Nevertheless, these signals underscore the need for careful perioperative monitoring and for future studies that specifically examine the safety profile of SGLT2 inhibitors in the immediate postoperative window.

The observed mortality benefit extends the cardiorenal protective effects of SGLT2 inhibitors documented in large randomized trials. In patients with heart failure with reduced ejection fraction, dapagliflozin and empagliflozin reduced the composite of cardiovascular death or heart failure hospitalization by 25–26% [7,8,17,18]. Similar benefits have been demonstrated in patients with preserved ejection fraction and chronic kidney disease [6,18]. Recent observational studies in patients undergoing TAVR have reported lower mortality and heart failure hospitalization with SGLT2 inhibitors [7,8,11], and meta-analyses in severe aortic stenosis populations undergoing TAVR have shown consistent reductions in all-cause mortality [12,19]. The present study broadens these observations to a wider spectrum of cardiac procedures, including surgical revascularization and valve interventions.

Multiple mechanisms may underlie these associations. SGLT2 inhibitors exert natriuretic and diuretic effects, improve myocardial energetics, reduce systemic inflammation, and attenuate oxidative stress, all pathways particularly relevant in the perioperative setting after cardiac surgery [13,14]. Preclinical and clinical data further suggest favorable effects on atrial remodeling and ventricular function that could contribute to improved long-term survival [9,10]. The reduction in acute kidney injury is consistent with the well-established renoprotective properties of this drug class [6,10,20].

Current perioperative guidelines from major societies (including the American Diabetes Association and several anesthesiology and surgical consensus statements) recommend withholding SGLT2 inhibitors for at least 3–4 days before elective surgery because of the risk of euglycemic diabetic ketoacidosis. Our findings must be interpreted strictly in this context. The exposure definition used in the present study reflects documented SGLT2-inhibitor use in the year preceding surgery; it does not indicate that therapy was continued through the perioperative period. Consequently, the observed associations with lower mortality and AKI cannot be taken as evidence that SGLT2 inhibitors should be continued up to the day of surgery. Rather, they suggest that patients who have been treated with these agents before surgery may derive lasting cardiorenal benefit. Prospective randomized trials that specifically evaluate the optimal timing of interruption and resumption of SGLT2-inhibitor therapy around major cardiac procedures are therefore needed before any change in perioperative practice can be recommended.

Strengths of this study include the large, contemporary, multi-institutional cohort derived from 66 U.S. healthcare organizations, rigorous 1:1 propensity score matching that achieved excellent balance across measured covariates, and evaluation of clinically relevant short- and long-term outcomes. The consistency of findings across diabetes-only and obesity-only subgroups and in landmark sensitivity analysis, together with the absence of association with falsification outcomes, supports the robustness of the results.

Nevertheless, limitations inherent to observational research must be acknowledged. Despite comprehensive propensity score matching, residual confounding by unmeasured factors cannot be excluded. Important perioperative variables that are known to influence postoperative outcomes, including operative urgency, surgical complexity, left-ventricular ejection fraction, frailty, cardiopulmonary-bypass time, surgeon experience, and validated operative risk scores, are not available in the TriNetX platform and therefore could not be balanced between groups. These unmeasured factors may have contributed to the observed differences in mortality and other endpoints. In addition, the cohort combined several distinct cardiac procedures. Procedure-specific subgroup analyses were not performed and would be required to determine whether the observed associations differ across CABG, surgical valve interventions, TAVR, and thoracic aortic procedures.

In addition, the database does not contain information on the precise timing of the last preoperative dose or on whether SGLT2 inhibitors were withheld and later resumed according to current perioperative recommendations; this absence of interruption and resumption data is an important limitation when interpreting the observed associations. Because exposure was defined entirely before the index date, classic post-index immortal time bias is not the primary concern. More relevant potential biases include exposure misclassification, prevalent-user bias, and healthy-user bias. The 30-day landmark analysis was performed to examine whether the observed associations persisted after exclusion of early postoperative events rather than to eliminate immortal time bias. TriNetX data do not capture medication adherence, dosing, or events occurring outside participating health systems. Finally, the study population reflects contemporary U.S. practice and may not be generalizable to lower-risk or non-U.S. cohorts.

## 5. Conclusions

In conclusion, SGLT2 inhibitor use was associated with lower all-cause mortality and acute kidney injury after major cardiac procedures in this large propensity score-matched cohort. These real-world data add to the growing body of evidence supporting the cardiovascular and renal protective effects of SGLT2 inhibitors and provide a strong rationale for dedicated prospective randomized trials evaluating perioperative SGLT2 inhibitor therapy in patients undergoing CABG, valve surgery, and TAVR.

## Figures and Tables

**Table 1:** Baseline Characteristics of the Study Cohorts Before and After Propensity Score Matching.

Variable	Before Matching Group 1 (SGLT2i) (n = 7625)	Before Matching Group 2 (Control) (n = 136,548)	Std. Diff.	After Matching Group 1 (SGLT2i) (n = 7526)	After Matching Group 2 (Control) (n = 7526)	Std. Diff.
Total Patients	7625	136,548	—	7526	7526	—
Age at Index (years), Mean ± SD	65.7 ± 10.7	67.7 ± 12.2	0.169	65.8 ± 10.6	66.1 ± 11.3	0.025
Male, n (%)	5388 (70.7)	87,147 (67.1)	0.077	5305 (70.5)	5323 (70.7)	0.005
Female, n (%)	2237 (29.3)	49,401 (36.2)	—	2221 (29.5)	2203 (29.3)	—
White race, n (%)	5853 (76.8)	108,010 (83.2)	0.161	5788 (76.9)	5831 (77.5)	0.014
Diabetes mellitus, n (%)	5858 (76.8)	38,065 (29.3)	1.083	5759 (76.5)	5871 (78.0)	0.036
Essential hypertension, n (%)	6115 (80.2)	86,822 (66.9)	0.306	6029 (80.1)	6039 (80.2)	0.003
Chronic kidney disease, n (%)	2072 (27.2)	18,072 (13.9)	0.333	2021 (26.9)	2029 (27.0)	0.002
Acute myocardial infarction, n (%)	2074 (27.2)	27,603 (21.3)	0.330	2689 (35.7)	2707 (36.0)	0.005
Heart failure, n (%)	4121 (54.0)	36,035 (27.7)	0.555	4023 (53.5)	3944 (52.4)	0.021
Beta blockers, n (%)	6282 (82.4)	72,541 (55.9)	0.599	6183 (82.2)	6230 (82.8)	0.016
Statins, n (%)	6748 (88.5)	78,333 (60.3)	0.682	6650 (88.4)	6778 (90.1)	0.055
ACE inhibitors, n (%)	3162 (41.5)	34,353 (26.5)	0.321	3088 (41.0)	3232 (42.9)	0.039
BMI (kg/m^2^), Mean ± SD	30.7 ± 6.4	29.4 ± 6.1	0.215	30.7 ± 6.4	31.0 ± 6.4	0.050
eGFR (mL/min/1.73 m^2^), Mean ± SD	72.2 ± 26.3	73.4 ± 23.6	0.047	72.3 ± 26.2	70.3 ± 26.3	0.074
HbA1c (%), Mean ± SD	7.4 ± 1.7	6.2 ± 1.4	0.769	7.3 ± 1.7	7.3 ± 1.9	0.016

**Table 2:** Relative Risks and Risk Differences for Clinical Outcomes in Patients with versus without SGLT2i Use (Propensity Score-Matched Cohorts, n = 7526 per group).

Outcome	Timepoint	With SGLT2i n (%)	Without SGLT2i n (%)	RD (95% CI)	RR (95% CI)	*p* Value
All-cause mortality	30 days	99 (1.3)	175 (2.3)	−0.010 (−0.014, −0.006)	0.566 (0.443–0.722)	<0.001
	90 days	164 (2.2)	255 (3.4)	−0.012 (−0.017, −0.007)	0.643	<0.001
	1 year	285 (3.8)	434 (5.8)	−0.020 (−0.027, −0.013)	0.657	<0.001
MACE	30 days	1745 (23.2)	1795 (23.9)	−0.007 (−0.020, 0.007)	0.972	0.337
	90 days	2042 (27.1)	2078 (27.6)	−0.005	0.983	0.510
	1 year	2379 (31.6)	2449 (32.5)	−0.009	0.971	0.222
Hospital Admissions	30 days	5748 (76.4)	5173 (68.7)	0.076 (0.062, 0.091)	1.111 (1.089–1.133)	<0.001
Stroke	30 days	271 (3.6)	296 (3.9)	−0.003	0.916	0.285
Acute Kidney Injury	30 days	1157 (15.4)	1330 (17.7)	−0.023	0.870	<0.001
Hypotension	30 days	1463 (19.4)	1243 (16.5)	0.029	1.177	<0.001
Cardiac Arrest	30 days	116 (1.5)	145 (1.9)	−0.004	0.800	0.070
Ventricular Arrhythmia	30 days	423 (5.6)	300 (4.0)	0.016	1.410	<0.001
Cardioversion	30 days	85 (1.1)	81 (1.1)	0.001	1.049	0.755

**Table 3:** Subgroup Analysis: Association of SGLT2i Use with All-Cause Mortality at 1 Year (Diabetes-only and Obesity-only Subgroups).

Subgroup	SGLT2i Users Events (%)	Non-Users Events (%)	RD (95% CI)	RR (95% CI)	*p* Value
Diabetes-only	212 (4.1)	318 (6.2)	−0.021	0.66 (0.55–0.79)	<0.001
Obesity-only	68 (3.2)	109 (5.1)	−0.019	0.62 (0.46–0.84)	0.002

Separate 1:1 propensity score matching performed within each subgroup. Results are directionally consistent with the main analysis.

**Table 4:** Sensitivity Analysis: 30-Day Landmark Analysis (Outcomes from Day 31 to 1 Year).

Outcome	HR (95% CI)	*p* Value
All-cause mortality	0.64 (0.54–0.75)	<0.001
MACE	0.96 (0.91–1.02)	0.18
Stroke	0.85 (0.74–0.98)	0.026

## Data Availability

The data that support the findings of this study were obtained from the TriNetX U.S. Collaborative Network. These data are available to authorized users of the TriNetX platform, but restrictions apply to their availability. Data are not publicly available due to licensing restrictions and privacy requirements of the federated research network. Access to the data may be obtained by contacting TriNetX through an institutional membership.

## Supplementary Materials

The following supporting information can be downloaded at: https://www.mdpi.com/article/10.3390/jcm15176808/s1, Supplemental Table S1: Query Criteria for Cohort 1 (SGLT2i Group); Supplemental Table S2: Query Criteria for Cohort 2 (Control Group); Supplemental Table S3: Outcome Definitions; Supplemental Table S4: Propensity Score Matching; Supplemental Table S5: E Values, Absolute Risk Difference, and Relative Risk Reduction for Outcomes.

## Abbreviations

**Abbreviation**	**Full Term**
AKI	Acute kidney injury
BMI	Body mass index
CABG	Coronary artery bypass grafting
CI	Confidence interval
eGFR	Estimated glomerular filtration rate
HbA1c	Hemoglobin A1c
HR	Hazard ratio
MACE	Major adverse cardiovascular events
PSM	Propensity score matching
RD	Risk difference
RR	Risk ratio
SD	Standard deviation
SGLT2	Sodium–glucose cotransporter-2
SGLT2i	Sodium–glucose cotransporter-2 inhibitor(s)
SMD	Standardized mean difference
TAVR	Transcatheter aortic valve replacement
