# Supplementary material for: Preoperative SGLT2 Inhibitor Use and Outcomes After Major Cardiac Procedures: A Propensity Score-Matched Analysis of the TriNetX Network
Source: J Clin Med. 2026 Sep 2;15(17):6808. doi: 10.3390/jcm15176808 (PMC13565910; doi:10.3390/jcm15176808)
Supplement: Supplementary file 1 [file jcm-15-06808-s001.zip › jcm-4448091-supplementary.pdf]

APPENDIX

Supplemental Table S1: Query Criteria for Cohort 1 (SGLT2i Group)

Supplemental Table S2: Query Criteria for Cohort 2 (Control Group)

Supplemental Table S3: Outcome Definitions

Supplemental Table S4: Propensity Score Matching

Supplemental Table S5: E Values, Absolute Risk Difference, and Relative Risk Reduction for Outcomes

Supplemental Table S1: Query Criteria for Cohort 1 (SGLT2i Group)

Ungrouped terms					
	must have		demographics	Age	Age (at least 18 years (most recent occurrence))
Group 1					
	**Group 1A**				
	must have	any of	procedure	UMLS:CPT:1006217	Coronary artery bypass, using arterial graft(s)
			procedure	UMLS:CPT:1006200	Coronary artery bypass, vein only
			procedure	UMLS:CPT:33405	Replacement, aortic valve, open, with cardiopulmonary bypass; with prosthetic valve other than homograft or stentless valve
			procedure	UMLS:CPT:1021150	Transcatheter aortic valve replacement (TAVR/TAVI) with prosthetic valve
			procedure	UMLS:ICD10PCS:02RX	Heart And Great Vessels / Replacement / Thoracic Aorta, Ascending/arch
			procedure	UMLS:CPT:1006159	Surgical Procedures on the Mitral Valve
			procedure	UMLS:ICD10PCS:02QX	Heart And Great Vessels / Repair / Thoracic Aorta, Ascending/arch
	date constraint		The terms in this group occurred at any time		
	event relationship		Any instance of Group 1B occurred at least 1 day before any instance of Group 1A		
	**Group 1B**				
	cannot have		diagnosis	UMLS:ICD10CM:I48	Atrial fibrillation and flutter
		or	diagnosis	UMLS:ICD10CM:Z94.1	Heart transplant status
		or	diagnosis	UMLS:ICD10CM:N18.6	End stage renal disease
		or	procedure	UMLS:ICD10PCS:02HA0QZ	Insertion of Implantable Heart Assist System into Heart, Open Approach
		or	diagnosis	UMLS:ICD10CM:Q20-Q28	Congenital malformations of the circulatory system
Group 2					
	**Group 2A**				
	must have	any of	procedure	UMLS:CPT:1006217	Coronary artery bypass, using arterial graft(s)
			procedure	UMLS:CPT:1006200	Coronary artery bypass, vein only
			procedure	UMLS:CPT:33405	Replacement, aortic valve, open, with cardiopulmonary bypass; with prosthetic valve other than homograft or stentless valve
			procedure	UMLS:CPT:1021150	Transcatheter aortic valve replacement (TAVR/TAVI) with prosthetic valve
			procedure	UMLS:ICD10PCS:02RX	Heart And Great Vessels / Replacement / Thoracic Aorta, Ascending/arch

			procedure	UMLS:CPT:1006159	Surgical Procedures on the Mitral Valve
			procedure	UMLS:ICD10PCS:02Q X	Heart And Great Vessels / Repair / Thoracic Aorta, Ascending/arch
	date constraint		The terms in this group occurred at any time		
	event relationship		Any instance of Group 2B occurred within 1 year and 1 day before any instance of Group 2A		
	**Group 2B**				
	must have		medication	NLM:ATC:A10BK	Sodium-glucose co-transporter 2 (SGLT2) inhibitors

Supplemental Table S2: Query Criteria for Cohort 2 (Control Group)

Ungrouped terms					
	must have		demographics	Age	Age (at least 18 years (most recent occurrence))
Group 1					
	**Group 1A**				
	must have	any of	procedure	UMLS:CPT:1006217	Coronary artery bypass, using arterial graft(s)
			procedure	UMLS:CPT:1006200	Coronary artery bypass, vein only
			procedure	UMLS:CPT:33405	Replacement, aortic valve, open, with cardiopulmonary bypass; with prosthetic valve other than homograft or stentless valve
			procedure	UMLS:CPT:1021150	Transcatheter aortic valve replacement (TAVR/TAVI) with prosthetic valve
			procedure	UMLS:ICD10PCS:02RX	Heart And Great Vessels / Replacement / Thoracic Aorta, Ascending/arch
			procedure	UMLS:CPT:1006159	Surgical Procedures on the Mitral Valve
			procedure	UMLS:ICD10PCS:02QX	Heart And Great Vessels / Repair / Thoracic Aorta, Ascending/arch
	cannot have		medication	NLM:ATC:A10BK	Sodium-glucose co-transporter 2 (SGLT2) inhibitors
	date constraint		The terms in this group occurred at any time		
	event relationship		Any instance of Group 1B occurred at least 1 day before any instance of Group 1A		
	**Group 1B**				
	cannot have		diagnosis	UMLS:ICD10CM:I48	Atrial fibrillation and flutter
		or	diagnosis	UMLS:ICD10CM:Z94.1	Heart transplant status
		or	diagnosis	UMLS:ICD10CM:N18.6	End stage renal disease
		or	procedure	UMLS:ICD10PCS:02HA0QZ	Insertion of Implantable Heart Assist System into Heart, Open Approach
		or	diagnosis	UMLS:ICD10CM:Q20-Q28	Congenital malformations of the circulatory system

Supplemental Table S3: Outcome Definitions

Deceased/All-Cause Mortality			
**Outcome definition**			
	Demographics	Deceased	Deceased
**Settings for the performed analyses**			
	Risk analysis		including patients with outcome prior to the time window
	Kaplan - Meier survival analysis		including patients with outcome prior to the time window
MACE			
**Outcome definition**			
	Demographics	Deceased	Deceased
	Diagnosis	UMLS:ICD10CM:I21	Acute myocardial infarction
	Diagnosis	UMLS:ICD10CM:I63	Cerebral infarction
**Settings for the performed analyses**			
	Risk analysis		including patients with outcome prior to the time window
	Kaplan - Meier survival analysis		including patients with outcome prior to the time window
Admissions			
**Outcome definition**			
	Visit	UMLS:HL7V3.0:VisitType:IMP	Visit: Inpatient Encounter
	Visit	UMLS:HL7V3.0:VisitType:NONAC	Visit: Inpatient Non-acute
**Settings for the performed analyses**			
	Risk analysis		including patients with outcome prior to the time window
	Kaplan - Meier survival analysis		including patients with outcome prior to the time window
Stroke			
**Outcome definition**			
	Diagnosis	UMLS:ICD10CM:I63	Cerebral infarction
**Settings for the performed analyses**			
	Risk analysis		including patients with outcome prior to the time window
	Kaplan - Meier survival analysis		including patients with outcome prior to the time window
AKI			
**Outcome definition**			
	Diagnosis	UMLS:ICD10CM:N17	Acute kidney failure
**Settings for the performed analyses**			
	Risk analysis		including patients with outcome prior to the time window
	Kaplan - Meier survival analysis		including patients with outcome prior to the time window
Hypotension			
**Outcome definition**			
	Diagnosis	UMLS:ICD10CM:I95	Hypotension
**Settings for the performed analyses**			
	Risk analysis		including patients with outcome prior to the time window
	Kaplan - Meier survival analysis		including patients with outcome prior to the time window

Cardiac Arrest			
	**Outcome definition**		
	Diagnosis	UMLS:ICD10CM:I46	Cardiac arrest
	**Settings for the performed analyses**		
	Risk analysis		including patients with outcome prior to the time window
	Kaplan - Meier survival analysis		including patients with outcome prior to the time window
Ventricular Arrhythmia			
	**Outcome definition**		
	Diagnosis	UMLS:ICD10CM:I47.2	Ventricular tachycardia
	Diagnosis	UMLS:ICD10CM:I49.0	Ventricular fibrillation and flutter
	**Settings for the performed analyses**		
	Risk analysis		including patients with outcome prior to the time window
	Kaplan - Meier survival analysis		including patients with outcome prior to the time window
Cardioversion			
	**Outcome definition**		
	Procedure	UMLS:CPT:92960	Cardioversion, elective, electrical conversion of arrhythmia; external
	Procedure	UMLS:CPT:92961	Cardioversion, elective, electrical conversion of arrhythmia; internal (separate procedure)
	**Settings for the performed analyses**		
	Risk analysis		including patients with outcome prior to the time window
	Kaplan - Meier survival analysis		including patients with outcome prior to the time window

Supplemental Table S4: Propensity Score Matching

**Cohort 1 and cohort 2 patient count before and after propensity score matching**			
	Cohort	Patient count before matching	Patient count after matching
	1 - - TAVR Main Intervention Group	7,625	7,526
	2 - TAVR Main Control Group	136,348	7,526

Propensity score density function - Before and after matching (cohort 1 - purple, cohort 2 - green)

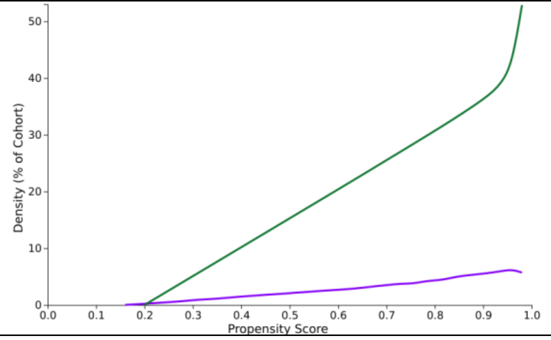

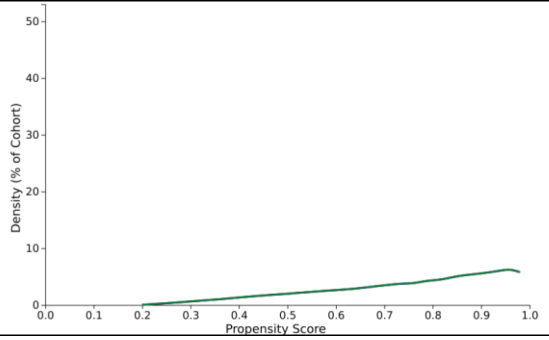

Supplemental Table S5: E Values, Absolute Risk Difference, and Relative Risk Reduction for Outcomes

Outcomes are compared for a follow-up period of up to 1 year. Absolute risk difference (ARD) represents the difference in event rates between SGLT2i users and non-users. Relative risk reduction (RRR) was calculated from the corresponding risk ratios.

Outcome	E-value HR	E-value CI	Absolute Risk Difference	Relative Risk Reduction
**All-cause mortality (1 year)**	**2.45**	**2.05**	**−2.0%**	**34.3%**
**MACE (1 year)**	**1.15**	**1.00**	**−0.9%**	**2.9%**
**Acute kidney injury (30 d)**	**1.45**	**1.25**	**−2.3%**	**13.0%**
